# Relationship Between Violence Against Children and Dating Violence Patterns Among Brazilian Adolescents: A Latent Class Analysis

**DOI:** 10.1007/s40653-026-00939-7

**Published:** 2026-07-03

**Authors:** Claudia Leite de Moraes, Daniela Porto Faus, Michael Eduardo Reichenheim, Marcia C. Castro, Stella Regina Taquette

**Affiliations:** 1 Institute of Social Medicine Hesio Cordeiro, Rio de Janeiro State University (IMS/UERJ), Rua São Francisco Xavier, 524 / 7º andar, Bloco D, Maracanã, Rio de Janeiro, RJ 20550013 Brasil; 2 Maternity-School hospital, Federal University of Rio de Janeiro (UFRJ), Rua das Laranjeiras, 180 - Laranjeiras, Rio de Janeiro, RJ 22240-00 Brasil; 3 Department of Global Health and Population, Harvard TH Chan School of Public Health, 655 Huntington Ave, Boston, MA 02115 USA; 4 Faculty of Medical Sciences, Rio de Janeiro State University (FCM/UERJ), Avenida Professor Manuel de Abreu, 444, Maracanã, Rio de Janeiro, RJ 20550-170 Brasil

**Keywords:** Child Abuse, Dating Violence, Adolescence, Latent Class Analysis, Brazil

## Abstract

Studies examining the associations between violence against children (VAC) and patterns of adolescent dating violence (DV) perpetration and victimization using person-centered analytic approaches remain limited and have yielded inconsistent findings. This study addresses whether emotional, physical, and sexual violence against children (VAC) are risk factors for distinct patterns of adolescent dating violence (DV), considering adolescents’ gender and their roles as victims or perpetrators. This is a school based cross-sectional study that involved a stratified cluster sample of 550 s-year high school students from thirteen schools in Rio de Janeiro, Brazil. The Childhood Trauma Questionnaire (CTQ) was used to detect VAC, while DV was identified using the Brazilian version of the Conflict in Adolescent Dating Relationships Inventory (CADRI). Latent Class Analysis (LCA) was used to identify classes of individuals based on their response patterns to the CADRI items. Multinomial regression analysis examined the relationships between VAC and DV patterns. Among girls, no single type of VAC increased the odds of belonging to the most severe perpetration class of DV. However, childhood sexual abuse increased fivefold (OR: 5.72; 95% CI: 2.51–13.06) the likelihood of belonging to the most severe DV victimization class. Among boys, childhood emotional (OR: 1.65; 95% CI: 1.65 - 16.57) and physical abuse (OR: 2.66; 95% CI: 1.01 - 7.05) were associated with higher odds of DV perpetration. Emotional VAC increased boys’ likelihood of belonging to the poly-victimization class (OR: 5.97; 95% CI: 1.95 - 18.30). Childhood physical abuse also increased their likelihood of belonging to severe class by more than three-fold (OR: 3.60; 95% CI: 1.80–7.21). The effects of childhood victimization on adolescent DV differ by sex, type of childhood violence, and whether the adolescent is a perpetrator or victim. These findings underscore the need for sex-specific and trauma-informed strategies to prevent dating violence during adolescence.

## Introduction

### Background

Romantic relationships play a central role in adolescent development, contributing to identity formation and shaping attachment patterns. However, they can also be marked by conflict and violence (Wolfe & Temple, 2018). Although estimates of dating violence (DV) vary widely across studies, literature consistently highlights its substantial magnitude. A comprehensive meta-analysis conducted across multiple continents found that approximately 20% of adolescents reported involvement in romantic relationships characterized by physical violence, and 9% reported sexual violence. Clear sex differences emerged: a significantly higher proportion of female adolescents (25%) reported perpetrating physical violence against a dating partner compared with males (9%), whereas a larger proportion of males (10%) reported perpetrating sexual dating violence compared with females (3%) (Wincentak et al., 2017). A more recent systematic review focusing on European studies further reinforced both the high prevalence of DV and the considerable variability by sex. For psychological victimization, prevalence ranged from 6% to 95.5% among females and from 5.6% to 94.5% among males. Physical victimization ranged from 2.2% to 33% among females and from 0.8% to 30% among males. Sexual victimization ranged from 5% to 41% among females and from 2.4% to 39% among males (Tomaszewska & Schuster, 2021).

The relevance and complexity of the problem are further underscored when its consequences are considered. Beyond immediate physical consequences such as bruises, fractures, and other injuries, DV can lead to a wide range of adverse outcomes, including sexually transmitted infections, unintended pregnancies, and mental health problems, as well as increased substance use (Exner-Cortens et al., 2013; Wolfe & Temple, 2018). Experiencing violence during adolescence has also been shown to increase the likelihood of both victimization and perpetration in subsequent intimate relationships (Exner-Cortens et al., 2013; Wolfe & Temple, 2018). Furthermore, a recent review highlights the interconnected nature of dating violence with other forms of violence occurring across multiple contexts, including the family, school, community, and social media (Taquette & Monteiro, 2019). These wide-ranging consequences underscore the public health relevance of DV and reinforce the need to identify risk and protective factors to inform effective prevention strategies.

Violence against children (VAC) is also a major public health problem affecting millions of individuals worldwide (World Health Organization, 2024a, 2024b). Its high prevalence in South America underscores the magnitude of this phenomenon in the region (Faus et al., 2019; Stoltenborgh et al., 2015). Beyond its widespread occurrence, VAC has been consistently associated with adverse outcomes across the life course, including impairments in physical and mental health, in cognitive and educational development, as well as an increased vulnerability to other forms of violence in later stages of life (Fang & Corso, 2007; Faus et al., 2019; Hecker et al., 2016; Krug et al., 2002). Building evidence of the long-term consequences of VAC, several authors have suggested that exposure to violence in childhood may also increase the risk of intimate partner violence during adolescence and adulthood (Fang & Corso, 2007; Richards et al., 2017; Tussey et al., 2018).

Unfortunately, much of the existing literature has focused predominantly on girls/women as victims and boys/men as perpetrators, notwithstanding evidence indicating comparable and, in some cases, higher rates of female-perpetrated physical violence (Wolfe & Temple, 2018). The potential effect modification of sex in the association between VAC and DV also remains insufficiently explored and subject to debate. Some studies support the notion of sex symmetry, suggesting that the repercussions of early violence may be similar for girls/women and boys/men (McMahon et al., 2015). Others argue that the impact of childhood violence on DV differs between sexes (Varlıoğlu & Hayes, 2022). For example, a recent meta-analysis found that boys who experienced maltreatment during childhood were more likely than girls to perpetrate intimate partner violence in adulthood (Varlıoğlu & Hayes, 2022), whereas another study reported that maltreated girls, like maltreated boys, also face an elevated risk of violence perpetration later in life (Li et al., 2020).

Authors who argue that the negative health consequences of child abuse vary by sex emphasize the need to examine the association between VAC and DV stratified by sex and by adolescents’ roles in violent situations. This suggestion is supported by literature that indicates that girls who experience abuse more frequently develop internalizing symptoms, such as anxiety and depression, whereas boys tend to exhibit externalizing problems, including aggressiveness and the use of alcohol and illicit drugs (Curran et al., 2016). These distinct developmental trajectories may, in turn, shape different patterns of involvement in dating violence, with internalizing responses potentially increasing vulnerability to victimization and externalizing responses increasing the likelihood of perpetration. Therefore, according to those authors, analyses of the consequences of VAC should consider boys and girls separately and explicitly account for adolescents’ roles in DV (Pereda et al., 2022).

Sociodemographic characteristics and family context are also considered relevant factors when studying the association between VAC and DV (Russell & Voith, 2025). Particularly in low- and middle-income countries, low maternal educational attainment and black and mixed race/skin color, commonly used proxies for family socioeconomic status, are considered potential confounders when focusing in this relationship, as they are consistently associated with the occurrence of childhood violence and constitute risk factors for dating violence (Krug et al., 2002; Stoltenborgh et al., 2015). Family structure during childhood is also relevant, as children who do not live with both parents may be exposed to higher levels of family violence due to increased caregiver burden, low socioeconomic conditions and low social support (Krug et al., 2002). Reduced parental supervision, which is more frequent in single-parent households, may further increase adolescents’ involvement in dating violence (Rothman, 2018).

Recent literature on this relationship also highlighted limitations of classifying DV solely by the type of violence (e.g., physical, sexual, or psychological), as behaviors within the same category may vary substantially in their underlying dynamics, motivations, and consequences. To address this heterogeneity, person-centered approach, most notably latent class analysis (LCA), have been increasingly used to identify distinct patterns of DV. A systematic review of 20 studies published between 2000 and 2022 applying these methods consistently identified three to four latent classes characterized by different combinations of violent behaviors, underscoring the frequent co-occurrence of multiple forms of DV (Bellemare-Lepage et al., 2025). Using LCA, Pereda et al. (2022) reported associations between childhood corporal punishment and class membership reflecting both victimization and perpetration of controlling and physical DV. Similarly, Paradis et al. (2024) found that membership in classes characterized by psychological violence and control, as well as multiple forms of DV, was associated with exposure to traumatic experiences during childhood.

### The Current Study

The gaps identified in the literature highlight the need for further research on the associations between different forms of childhood victimization and patterns of adolescent dating violence. Studies must more thoroughly examine sex-specific differences in both victimization and perpetration. Advancing this understanding is essential for informing the development of effective, targeted interventions. Accordingly, this study aims to contribute to the existing literature by assessing whether emotional, physical, and sexual child victimization are risk factors for distinct classes of dating violence perpetration and victimization among adolescents, while considering potential differences between boys and girls.

## Methods

### Study Setting

The study was conducted among second-year high school students enrolled in public and private schools located in the IX Administrative Region (IX-AR) of the municipality of Rio de Janeiro, Brazil. The IX-AR is a densely populated urban area marked by substantial socioeconomic diversity and an estimated population of approximately 190,000 residents. The region exhibits pronounced inequalities in housing and living conditions, encompassing both affluent neighborhoods and underserved settlements known as favelas or comunidades. These areas represent irregular forms of land occupation and are typically characterized by limited urban infrastructure, restricted access to essential public services, and location in zones not formally designated for residential use (Instituto Pereira Passos, 2020).

### Participants

Study participants were selected using a stratified, multistage cluster sampling design. Stratification included three groups: private schools, public schools with daytime classes, and public schools with evening classes. This three-tiered approach was adopted because these subgroups differ in socioeconomic status, average age, and educational trajectories (factors known to influence the risk of DV).

A total of 26 classes from 13 schools, comprising 749 students, were selected with probability proportional to school size. All students in the selected classes were invited to participate, and refusals were minimal (3.7%; *n* = 28). Among the 721 students who consented, most (96.1%; *n* = 693) were adolescents aged 10–19 years. Of these, 550 (79.4%) reported having had a dating partner in the preceding 12 months and were therefore eligible for inclusion in this analysis.

### Fieldwork

Data collection occurred between September 2016 and February 2017. Students completed a comprehensive, self-administered questionnaire in their classrooms under the supervision of a trained fieldwork coordinator. Completion time averaged approximately 50 min. No financial incentives were provided for participation.

### Measurements

#### Dating Violence

The primary outcome for this study was a three-level classification of adolescents’ involvement in dating violence (DV), derived through Latent Class Analysis (LCA) using items from the Portuguese version of the *Conflict in Adolescent Dating Relationships Inventory* (CADRI) (Minayo et al., 2011; Wolfe et al., 2001). The CADRI is a validated self-report instrument composed of 25 items covering five dimensions: Verbal/Emotional Abuse (10 items), Physical Abuse (4 items), Relational Abuse (3 items), Sexual Abuse (4 items), and Threatening Behavior (4 items). Responses are provided on a four-level ordinal scale (“often,” “sometimes,” “seldom,” “never”) referring to the previous 12 months. Participants reported both victimization and perpetration. The CADRI was selected due to its comprehensive assessment of multiple DV forms and roles, and its strong psychometric properties (Exner-Cortens et al., 2016; Reichenheim et al., 2022; Tomaszewska & Schuster, 2021).

The outcome variable was derived using latent class analysis (LCA) to identify homogeneous groups of adolescents based on similar response patterns across the 25 items of the CADRI. LCA is an individual-centered analytic technique that uncovers unobserved (latent) groups based on shared response profiles and is particularly useful for multidimensional phenomena such as DV (Nylund-Gibson & Choi, 2018; Wang & Wang, 2012). The approach assumes that associations among a set of observed categorical variables (each item of the CADRI) can be explained by a finite number of mutually exclusive classes.

Latent class analysis was deliberately applied to characterize the outcome (patterns of dating violence) because the core objective of the main study was to capture the underlying structure and co-occurrence of multiple forms of intimate violence within dating relationships, which are not adequately represented by single indicators or additive scores. Modeling dating violence as a latent construct allowed us to identify empirically derived, homogeneous groups of adolescents characterized by distinct profiles of perpetration and victimization, thereby preserving the multidimensional nature of this phenomenon.

Analyses employed robust maximum likelihood (Muthén & Muthén, 2013). Solutions with one to six classes were estimated and compared using the Vuong–Lo–Mendell–Rubin (VLMR) likelihood ratio test, Akaike Information Criterion (AIC), Bayesian Information Criterion (BIC), and entropy. Higher entropy values (range 0–1) indicated clearer class separation. Model selection also considered conceptual interpretability and substantive plausibility (Nylund-Gibson & Choi, 2018).

Across both sexes and roles (victimization and perpetration), the three-class model offered the best fit. Class labels were assigned based on the predominant response probability profiles. For both boys and girls, Class 1 was characterized by involvement in a limited set of emotionally abusive behaviors and was labeled **“mild emotional violence.”** Among girls, perpetration-related Class 2 reflected **“intense emotional violence with deprecation**,**”** while Class 3 additionally included physical violence and sexual coercion, labeled **“intense emotional violence with deprecation**,** physical violence**,** and sexual coercion.”** Victimization classes among girls showed higher severity: Class 2 already involved sexual coercion, and Class 3 further included defamation and overt rape. These were labeled **“intense emotional violence with deprecation and sexual coercion”** (Class 2) and **“intense emotional violence with deprecation**,** defamation**,** physical violence**,** and rape”** (Class 3).

Among boys, perpetration-related Class 3 mirrored the female profile, encompassing emotional, physical, and sexual violence. However, Class 2 did not include physical violence and was labeled **“intense emotional violence with deprecation and sexual coercion.”** For victimization, boys reported lower severity overall. Class 2 involved only emotional violence with deprecation, while Class 3 added physical violence and sexual coercion. These were labeled **“intense emotional violence with deprecation”** (Class 2) and **“intense emotional violence with deprecation**,** physical violence**,** and sexual coercion”** (Class 3). More detailed information on the characteristics of the identified classes among girls and boys, stratified by adolescents’ roles in the violent situation, is presented in Tables 1 and 2.

**Table 1:** Characteristics of the latent classes of dating violence among girls’ perpetration and victimization

Girls	Class	Label	Description
Perpetration	Class 1	*Mild emotional violence.*	Low to moderate frequency of a limited number of emotional violence behaviors, with emotional violence item-endorsement probabilities around 30%.
	Class 2	*Intense emotional violence with deprecation.*	High frequency of multiple emotional violence behaviors (endorsement probabilities ≥ 60%), including deprecation (≥ 25%), with minimal physical violence (≤ 5%) and virtually no sexual violence (≤ 1%)
	Class 3	*Intense emotional violence with deprecation*,* physical violence*,* and sexual coercion.*	Very high levels of emotional violence (endorsement probabilities 76–90%), including deprecation (≥ 50%), co-occurring with frequent physical violence (≥ 50%) and moderate sexual coercion (≈ 10–20%); rape was not reported.
Victimization	Class 1	*Mild emotional violence.*	Low to moderate frequency of a limited number of emotional violence behaviors, with emotional violence item-endorsement probabilities around 30%.
	Class 2	*Intense emotional violence with sexual deprecation and sexual coercion.*	High frequency of emotional violence (≥ 70%), including emotional deprecation (≥ 30%), with moderate to high levels of sexual coercion; physical violence was not reported.
	Class 3	*Intense emotional violence with deprecation*,* defamation*,* physical violence*,* and rape.*	High frequency of emotional violence, including deprecation (≥ 50%) and defamation (< 50%), together with physical violence (≈ 30%) and sexual violence (≥ 30%), including rape (≥ 15%).

**Table 2:** Characteristics of the latent classes of dating violence among boys’ perpetration and victimization

Boys	Class	Label	Description
Perpetration	Class 1	*Mild emotional violence.*	Low to moderate frequency of a small number of emotional violence behaviors (probabilities around 30%).
	Class 2	*Intense emotional violence with sexual deprecation and sexual coercion.*	High frequency of emotional violence (endorsement probabilities ≥ 70%), including emotional deprecation (> 30%), combined with moderate to high levels of sexual coercion (≈ 18%); physical violence was not reported (< 1%).
	Class 3	*Intense emotional violence with deprecation*,* physical violence*,* and sexual coercion.*	High frequency of emotional violence accompanied by physical violence (> 30%) and sexual coercion (> 20%); rape was not reported.
Victimization	Class 1	*Mild emotional violence.*	Low to moderate exposure to selected emotional violence behaviors. (< 30%)
	Class 2	*Intense emotional violence with deprecation.*	High frequency of emotional violence (> 40%) with deprecation (≈ 25%), without physical (> 10%) or sexual violence (> 10%).
	Class 3	*Intense emotional violence with deprecation*,* physical violence*,* and sexual coercion.*	High frequency of emotional violence, including deprecation (> 50%), together with physical violence (> 50%) and sexual coercion (> 33%); rape was not reported.

Considering the LCA-derived patterns of dating violence victimization and perpetration stratified by sex, four outcomes for the present study were defined: boys’ perpetration, boys’ victimization, girls’ perpetration, and girls’ victimization. For each outcome, adolescents were classified into one of three latent classes, modeled as nominal variables.

#### Violence Against Children

VAC was assessed using the Brazilian version of the Childhood Trauma Questionnaire (CTQ), which captures experiences of abuse occurring up to 10 years of age (Bernstein & Fink, 1998; Grassi-Oliveira et al., 2014). The CTQ is one of the most widely used retrospective instruments for identifying childhood abuse and neglect and has been validated for use among adolescents, young adults, and adults (Pietrini et al., La valutazione retrospettiva del trauma infantile: una revisione degli strumenti., 2010). The questionnaire includes 25 items divided into five subscales: emotional abuse, physical abuse, sexual abuse, physical neglect, and emotional neglect, plus three validity items that assist in data quality control.

In this study, only the emotional, physical, and sexual abuse subscales were used. These subscales demonstrate strong internal consistency in the original instrument, with Cronbach’s alpha coefficients of 0.87, 0.83, and 0.92, respectively (Bernstein et al., 2003). Items are rated on a five-point Likert scale (“never,” “a few times,” “sometimes,” “many times,” “always”). For the present analyses, any response from “a few times” to “always” was treated as a positive endorsement. Participants who endorsed at least one item within a subscale were classified as positive for that type of abuse. Also, a cumulative child abuse score was computed ranging from 0 (no reported abuse) to 3 (emotional, physical, and sexual abuse).

The exposures to childhood violence were modeled as observed variables to allow a direct and interpretable assessment of how each specific type of childhood violence is associated with the identified outcome patterns, which is essential for informing targeted preventive interventions.

#### Sociodemographic and Family Composition

Students were categorized into three age groups: 15–16 years, 17 years, and 18–19 years. Ethnicity/skin color was self-classified into five categories, as defined by the Brazilian Institute of Geography and Statistics: white, brown/mixed-race, black, Asian, and indigenous. The family’s economic condition was measured through the Brazilian Economic Classification Criteria (CCEB) – 2015 version (Associação Brasileira de Empresas de Pesquisa (ABEP), 2014). The criteria define broad economic classes based on ownership of durable goods, domestic employee contracts, educational level of the family head, and access to public services. Using a scoring system that weighs the number of items present in the household and other relevant characteristics, families are divided into A, B, C, D, and E economic classes, A being the class with the highest and E with the lowest purchasing power. Maternal schooling was categorized into three levels: up to five years, six to eight years, and nine years or more. Schools were categorized as public or private, considering the institution’s economic sponsor. Family structure was represented by a dichotomous variable: “live with both parents until age ten” (*yes/no*).

### Data Analysis

**Fig. 1:**
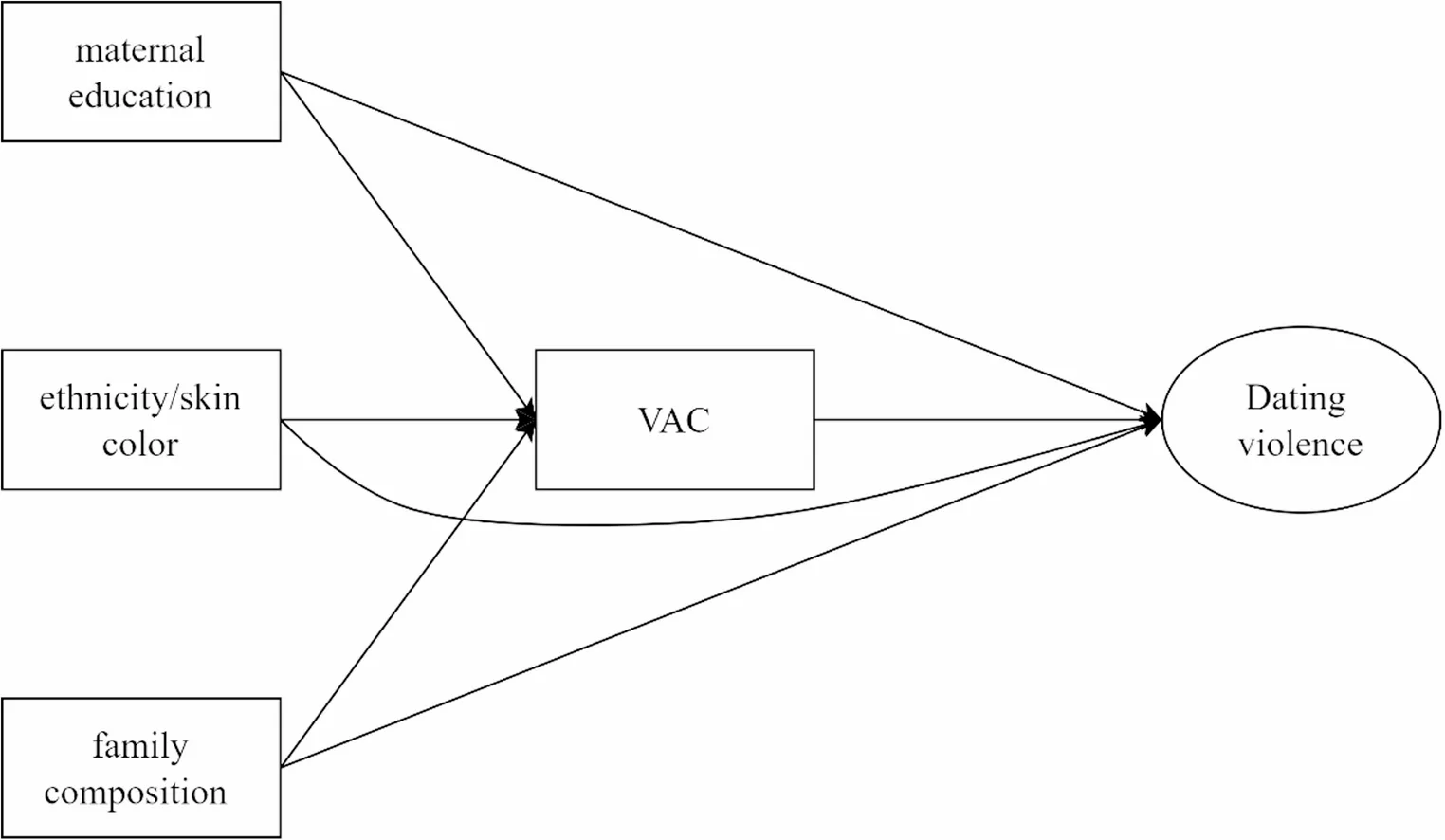
The minimal set of confounding variables necessary to obtain unbiased estimates of the associations between VAC and DV classes

We defined the minimal set of confounding variables necessary to obtain unbiased estimates of the associations between childhood violence dimensions and DV classes using DAGitty software (Textor et al., 2011). The final directed acyclic graph (DAG) is shown in Fig. 1.

Sixteen multinomial regression models were used to study the relationships between each exposure (emotional, physical, sexual, and cumulative violence during childhood) and the DV patterns among boys and girls (as perpetrators and victims, separately). Analyses were conducted separately for boys and girls to explore potential differences in the associations between violence against children (VAC) and dating violence patterns. These sex-stratified analyses were descriptive and comparative in nature, and no formal tests of interaction or effect modification by sex were performed.

Analyses followed the three-step procedure proposed by Choi et al. (2017), and Nylund-Gibson et al. (2018), which is recommended for appropriately accounting for measurement error. In the first step, we estimated the unconditional latent class model. In the second step, individuals were assigned to latent classes according to their highest posterior probability. In the final step, we estimated a mixture model with measurement parameters set to values that account for measurement error in the class assignment.

All analyses incorporated the complex sampling design (sample weights and clustering). Descriptive analyses were conducted in R version 4.0.4 (R. Core Team, 2021), and latent class analyses were performed using Mplus version 8 (Muthén & Muthén, 2013).

#### Ethics

All procedures followed the ethical standards of the institutional and national research committee and with the 1964 Helsinki Declaration and its later amendments or comparable ethical standards. The study was approved by the Bioethics Committee of the Rio de Janeiro State University (No. CAAE: 48107514.2.0000.5282) and by the State Secretariat of Education. AI tools were used during manuscript preparation to improve the translation from Portuguese into English and to assist with condensing the Conclusion section. All outputs were reviewed by the authors, who take full responsibility for the content of the manuscript.

## Results

### Sample Profile

The characteristics of the study population are presented in Table 3. The sample was predominantly composed of girls and adolescents aged 15–16 years, with most participants in socioeconomic class B. Indicators of vulnerability were present, including low maternal education and a substantial proportion not living with both parents during childhood. Exposure to childhood violence was frequent, particularly emotional (80.8%) and physical (55.8%) abuse, while sexual violence was less prevalent (11.1%), highlighting a clear gradient across types of violence.

**Table 3:** Sample profile according to demographic, socioeconomic, family composition and child abuse reports. School-based survey (IX-Administrative Region of the municipality of Rio de Janeiro, Brazil)

Variables		*N*	% [CI95%]
	Demographic		
Sex	Female	305	55.89 [55.82–55.96]
	Male	245	44.11 [44.04–44.18]
Age	15–16 years old	213	46.36 [46.30–46.42]
	17 years old	192	31.58 [31.52–31.63]
	18–19 years old	145	22.06 [22.01–22.10]
Ethnicity/Skin color	White	242	49.65 [49.57–49.73]
	Brown/Mestizo	195	32.37 [32.30–32.43]
	Black	93	15.64 [15.61–15.68]
	Indigenous	12	1.76 [17.50–17.70]
	Asian	5	0.58 [0.57–0.59]
	Socioeconomic		
Purchasing power of the families	A (wealthier families)	69	17.40 [17.34–17.45]
	B	263	54.50 [54.44–54.55]
	C	167	26.93 [26.89–26.96]
	D-E (poorer families)	9	1.18 [1.18–1.19]
Maternal education	≤ 5 years of schooling	225	38.49 [38.42–38.56]
	6 to 8 years of schooling	101	15.59 [15.56–15.63]
	> 8 years of schooling	193	45.92 [45.84–46.00]
School characteristic	Private	322	40.36 [40.31–40.41]
	Public	228	59.64 [59.59–59.69]
	Family structure		
Lived with both parents during childhood	No	150	27.90 [27.86–27.95]
	Yes	400	72.10 [72.05–72.14]
	History of childhood abuse		
Emotional	No	114	19.24 [19.19–19.29]
	Yes	431	80.76 [80.71–80.81]
Physical	No	234	44.22 [44.17–44.27]
	Yes	310	55.78 [55.73–55.83]
Sexual	No	491	88.88 [88.85–88.91]
	Yes	56	11.12 [11.09–11.15]

ª Estimates considering sample weights

### The Effects of Different Types of VAC Patterns Among Boys and Girls

The results of the multinomial regression models examining the associations between VAC and patterns of DV among boys and girls are presented in Table 4.

**Table 4:** Relationship between child abuse (exposures) and dating violence latent classes among adolescents (outcome). School-based survey (IX-Administrative Region of the municipality of Rio de Janeiro, Brazil)

	Child Abuse	*Perpetration*					*Victimization*				
		Class 1	Class 2		Class 3		Class 1	Class 2		Class 3	
		[34%]^***a***^	[39%] ^***a***^		[27%] ^***a***^		[43%] ^***a***^	[40%] ^***a***^		[17%] ^***a***^	
				OR_adj_^***b***^		OR_adj_^b^			OR_adj_^***b***^		OR_adj_^***b***^
Girls	**Emotional**	Ref	1.01	[0.39–2.61]	2.66	[0.73–9.73]	Ref	1.52	[0.71–3.25]	2.73	[0.73–10.18]
	**Physical**	Ref	0.98	[0.47–2.05]	1.36	[0.67–2.76]	Ref	1.44	[0.93–6.74]	2.50	[0.76–2.74]
	**Sexual**	Ref	2.23	[0.85–5.84]	1.77	[0.61–5.13]	Ref	1.02	[0.23–4.59]	5.72	[2.51–13.06]
	**Cumulative Violence**	Ref	1.17	[0.75–1.81]	1.45	[0.95–2.21]	Ref	1.27	[0.87–1.84]	2.66	[1.51–4.68]
		[48%] ^***a***^	[37%] ^***a***^		[15%] ^***a***^		[28%] ^***a***^	[49%] ^***a***^		[23%] ^***a***^	
				OR_adj_^***b***^		OR_adj_^***b***^			OR_adj_^***b***^		OR_adj_^***b***^
Boys	**Emotional**	Ref	0.86	[0.96 - 5.80]	1.65	[1.65 - 16.57]	Ref	7.45	[3.25 - 17.06]	5.97	[1.95 - 18.30]
	**Physical**	Ref	2.11	[0.94 - 4.72]	2.66	[1.01 - 7.05]	Ref	1.59	[0.77 - 3.29]	3.16	[1.07 - 9.34]
	**Sexual**	Ref	1.64	[0.34 - 7.92]	1.05	[0.14 - 7.59]	Ref	1.48	[0.18 - 12.37]	4.63	[0.65 - 32.95]
	**Cumulative Violence**	Ref	1.89	[1.19–3.01]	2.16	[1.32–3.55]	Ref	2.45	[1.50–4.00]	3.60	[1.80–7.21]

^a^ Between brackets: latent class prevalence;

^b^ Multinomial Odds Ratio adjusted for ethnicity/skin color, maternal education and living with both parents. 95% confidence intervals in brackets

#### The Relationship Between VAC and Perpetration Patterns of DV

Concerning the perpetration of DV, no single type of VAC increased the odds of belonging to the most severe classes (classes 2 and 3) of DV perpetration by girls when compared to the baseline (class 1). In turn, boys’ emotional (OR: 1.65; 95% CI: 1.65 - 16.57) and physical victimization during childhood (OR: 2.66; 95% CI: 1.01 - 7.05) were statistically associated with increased chances of belonging to class 3 of DV perpetration involving the practice of emotional, physical and sexual violence in romantic relationships.

#### The Relationship Between VAC and Victimization Patterns of DV

Considering DV victimization among girls, childhood sexual violence increased more than five-fold (OR: 5.72; 95% CI: 2.51–13.06) the probability of belonging to the most severe class of dating victimization compared to belonging to the reference class. In contrast, a history of emotional and physical abuse was not associated with pertaining to class 2 or 3. A different profile occurred among boys. While sexual victimization did not relate to DV, childhood emotional violence raised more than seven (OR: 7.45; 95% CI: 3.25 - 17.06) and six times (OR: 5.97; 95% CI: 1.95 - 18.30) the likelihood of adolescents being labeled as classes 2 and 3, respectively, when compared to belonging to class 1. Childhood physical abuse also increased their likelihood of belonging to the poly-victimization class (class 3) by more than three-fold (OR: 3.60; 95% CI: 1.80–7.21) when compared to pertaining to the reference class.

#### The Relationship Between Accumulation of VAC and DV

The addition of different types of VAC during childhood increased the probability of being classified in classes 2 and 3 as perpetrator and victim among boys. As perpetrator, the probability of pertaining to class 2 and 3 are 1.89 (CI: 95% 1.19–3.01) and 2.16 (CI: 1.32–3.55) higher than to pertaining to class 1, respectively. As victims, the accumulation of VAC also increased the probability of belonging to class 2 (2.45; 95% CI: 1.50–4.00) and class 3 (OR: 3.60; 95% CI: 1.80–7.21). In contrast, this accumulation only linearly increased the likelihood of belonging to the most severe classes of DV victimization among girls (OR: 2.66; 95% CI: 1.51–4.68).

## Discussion

The present study shows that all adolescents in the sample perpetrated or experienced some pattern of dating violence. In no instance did we identify a class characterized by the complete absence of violent behaviors, suggesting that dating relationships free from violence are highly uncommon among Brazilian adolescents. Even the least severe classes captured youth who were involved in emotional abuse, either as perpetrators or victims. These patterns underscore the urgent need for policies and interventions specifically tailored to prevent DV among adolescents in Brazill.

The study results indicate that emotional, physical, and sexual violence suffered during childhood increase the likelihood of involvement in DV during adolescence, particularly among adolescents classified in the poly-victimization profiles. These findings are consistent with previous latent class and person-centered studies examining the relationship between childhood adversity and adolescent dating violence patterns. Paradis et al. (2024), for example, identified a higher prevalence of childhood trauma among adolescents in the multiple dating violence class, supporting our finding that cumulative exposure to childhood violence is associated with more severe and overlapping dating violence profiles. However, it is important to note that their latent classes were derived from CADRI subscale scores rather than item-level indicators, and that their analyses were not stratified by sex, limiting the identification of sex-specific patterns of victimization and perpetration. In the same direction of our results, Pereda et al. (2022) found that corporal punishment and bullying at age 7 increased the likelihood of belonging to a “controlling and physical violence overlap” class at age 17, characterized by high probabilities of both perpetration and victimization involving controlling and physical violence. Notably, children exposed to corporal punishment were nearly twice as likely to both perpetrate and experience dating violence during adolescence.

Several theoretical perspectives shed light on the pathways linking childhood victimization to later IPV. Social learning theory (Bandura, 1978), the most widely cited among these frameworks, emphasizes how direct and indirect exposure to violence during childhood shapes cognitive models that normalize violent behaviors in relationships. Early experiences of violence may occur through verbal reinforcement, modeling, witnessing violence at home or in the community, or being directly victimized (Mattingly & Straus, 2008). These processes foster cognitive scripts that increase the probability of replicating violent behaviors in intimate relationships. Within a developmental-ecological framework, child abuse and neglect increase the risk of dating violence by disrupting attachment, emotion regulation, and social learning processes formed in early family environments, which are then reinforced across peer, community, and cultural contexts (Bronfenbrenner, 1979). General strain theory (Agnew, 1992) and the alcohol myopia model (Steele & Josephs, 1990), for instance, suggest that exposure to violence may lead individuals to cope with stress through substance use, which can increase susceptibility to aggression. According to Agnew (1992), adolescents may resort to alcohol or drugs to deal with the negative emotions associated with stressful experiences. Empirical studies reinforce that substance use may function as an attempt to regulate emotional distress or manage traumatic memories (Crimmins et al., 2000). The alcohol myopia model posits that acute intoxication narrows perceptual focus, heightening attention to provocative cues and weakening inhibitory control, thereby increasing the likelihood of aggressive responses (Steele & Josephs, 1990).

Given these theoretical underpinnings, it is crucial to consider sex differences in the pathways linking childhood abuse and DV. Our findings reinforce this point: the associations between childhood violence and DV patterns seems to vary by sex and by the type of abuse experienced as suggested by some of the previous studies (Varlıoğlu & Hayes, 2022). Among girls, none of the childhood violence indicators increased the likelihood of belonging to the most severe perpetration classes. In contrast, childhood sexual abuse markedly increased the probability of belonging to the most severe victimization class, which is characterized by multiple forms of psychological, physical and sexual abuse, including dating rape. This pattern suggests that girls exposed to sexual abuse early in life may be especially vulnerable to revictimization in adolescence. Feelings of guilt, diminished self-esteem, and perceived inadequacy, frequently reported among survivors of childhood sexual abuse, may contribute to their involvement in abusive romantic relationships during adolescence (Faus et al., 2019; Ihongbe & Masho, 2018).

In contrast, among boys, emotional and physical violence during childhood substantially increased the likelihood of belonging to the most severe DV perpetration and victimization classes. Although findings across studies are not entirely consistent (Holt et al., 2008), the literature generally shows that exposure to early-life violence has distinct mental health and behavioral impacts on boys and girls. Boys exposed to violence are more likely to develop externalizing behaviors, including aggression and violent conduct, whereas girls tend to show internalizing symptoms, such as emotional distress or depression (Curran et al., 2016; Varlıoğlu & Hayes, 2022). Evidence also suggests that male students who experience physical violence inside or outside the home are more likely than females to both approve of and engage in violent behaviors (Mattingly & Straus, 2008). Further, studies by Walters et al. (2020) and Garthe et al. (2019) show that parental acceptance of violence and peer support for fighting are more strongly associated with aggressive behavior among male adolescents. These mechanisms may help explain why boys who experienced emotional and physical violence in childhood were disproportionately concentrated in the most severe DV perpetration class during adolescence in our study. Supporting this interpretation, a qualitative study conducted with a subsample of our participants found that boys’ narratives centered more frequently on perpetrating violence, whereas girls emphasized their experiences as victims (Taquette et al., 2020).

Sex differences are also present in the associations between cumulative childhood victimization and DV patterns. Our findings show that boys exposed to multiple forms of childhood abuse were more likely to occupy the most severe patterns of both perpetration and victimization, whereas among girls, cumulative exposure was primarily associated with severe victimization only. Several authors highlight the importance of assessing exposure to multiple forms of violence during childhood, noting that such experiences rarely occur in isolation (Krug et al., 2002). Expanding this perspective, victims of child abuse may also be exposed to other adverse experiences, such as poverty, witnessing parental IPV, parental mental illness, incarceration, or divorce, all of which have well-established, long-term consequences for physical and mental health (Felitti et al., 1998). This growing body of evidence underscores the need for multidisciplinary, coordinated interventions to address VAC and DV. Preventive strategies should integrate efforts across families, schools, communities, and social institutions in order to mitigate these interconnected risks (Garthe et al., 2021).

Putting all together, our findings suggest that adolescent dating violence (DV) reflects interconnected patterns of perpetration and victimization, rather than distinct roles. The near absence of violence-free relationships and the presence of emotional abuse even in less severe classes indicate widespread, often overlapping involvement. Childhood violence emerges as a key shared determinant of both perpetration and victimization, although pathways probably differ by sex as boys exposed to early emotional and physical abuse are more likely to occupy severe profiles involving both perpetration and victimization, whereas girls, particularly those exposed to sexual abuse, are more concentrated in severe victimization patterns. These results highlight that DV roles are co-constructed through early-life experiences and developmental processes, reinforcing the need for integrated, gender-sensitive prevention strategies.

Our findings must be interpreted considering limitations and strengths. A recurrent concern in school-based surveys relates to truancy. Adolescents exposed to more severe forms of violence in childhood frequently exhibit academic difficulties and higher dropout rates, which makes it possible to be underrepresented in the sample (Faus et al., 2019). If these adolescents are indeed at heightened risk for DV, our estimates are likely conservative, suggesting that the true associations may be even stronger. Another limitation of the study relates to its cross-sectional design and the challenges of defining the temporal assumptions of causality. Nevertheless, we reinforce that the use of CTQ items anchored to exposure before age ten probably reduced concerns about temporal ordering. Another constraint is that retrospective self-reporting of childhood abuse may be subject to recall bias or underreporting, particularly for more sensitive experiences such as sexual violence, which could lead to misclassification and attenuated estimates. Despite these constraints, the consistency of the associations and their alignment with established theoretical frameworks support the plausibility of the identified links. We must also acknowledge that the study did not distinguish heterosexual from same-sex dating relationships. Prior evidence suggests that the dynamics of DV, including perpetration and victimization profiles, may differ by the gender configuration of the couple (Sulla et al., 2025). Future studies should consider this dimension to better understand how sexual orientation and gender identity interact with VAC and DV trajectories. Finally, the lack of formal moderation analyses was also a limitation. Although results were stratified by sex and role in violence (victim or perpetrator), this approach does not test whether associations differ significantly between groups. This decision was due to sample size constraints. Future studies with larger sample sizes should use multigroup analyses to formally assess this point.

Despite these limitations, the study offers also strengths. It encompasses a large, socioeconomically, and racially diverse region of Rio de Janeiro and includes students from both public and private schools. This diversity enhances the generalizability of our findings to other urban centers in Brazil and potentially to comparable low- and middle-income settings facing similar social inequalities. A further strength is the use of the Brazilian version of the CTQ, which, like the original instrument, clearly anchors exposure to events before the age of ten. This reduces concerns of temporal ambiguity between VAC and adolescent DV, a common challenge in cross-sectional designs. The application of latent class analysis represents another contribution. By adopting a person-centered analytical strategy, we identified distinct and meaningful patterns of DV that reflect the complexity of adolescent relationship dynamics. This approach is well suited for informing tailored prevention strategies targeting specific subgroups (Hébert et al., 2022). The decision to use item-level CADRI responses, rather than aggregated scores, further strengthened the precision and interpretability of the latent classes, allowing for a more robust examination of the VAC–DV relationship.

## Conclusion

Taken together, these findings demonstrate distinct sex-specific pathways linking childhood violence exposure to adolescent dating violence among Brazilian adolescents. Among girls, childhood sexual abuse was associated with more severe dating violence poly-victimization, whereas among boys, childhood emotional and physical victimization were associated with both victimization and perpetration of dating violence. These results reinforce the importance of trauma-informed and sex-specific prevention strategies, highlighting that efforts to prevent violence against children are essential to reducing adolescent dating violence. Specifically, early detection initiatives should prioritize girls exposed to sexual abuse and boys exposed to emotional and physical victimization, particularly within school and health-service settings, where adolescents can be identified and supported early.

## Data Availability

Not available.

## Declarations

### Use of Artificial Intelligence (AI)

AI tools were used during manuscript preparation to improve the translation from Portuguese into English and to assist with condensing the Conclusion section. All outputs were reviewed by the authors, who take full responsibility for the content of the manuscript.
